# Regional coordinators: a new teaching opportunity in family medicine training

**DOI:** 10.1186/s12909-016-0667-4

**Published:** 2016-05-10

**Authors:** Davorina Petek, Polona Vidič Hudobivnik, Viktorija Jančar, Bojana Petek, Zalika Klemenc-Ketiš

**Affiliations:** Department of Family Medicine, Faculty of Medicine, University of Ljubljana, Poljanski nasip 58, Ljubljana, 1000 Slovenia; Medical Centre Kočevje, Roška cesta 18, Kočevje, 1330 Slovenia; Health Centre Dr. Adolfa Drolca, Ulica talcev 9, Maribor, Slovenia; University Psychiatric Clinic Ljubljana, Studenec 48, Ljubljana, 1260 Slovenia; Department of Family Medicine, Maribor Medical School, University of Maribor, Taborska 8, Maribor, 2000 Slovenia

**Keywords:** Family practice, Education, Residency, Mentors, Qualitative research

## Abstract

**Background:**

A new project on education in family medicine training was implemented last year in Slovenia by establishing regional coordinators in the specialist training programme. They are responsible for conducting regular small-group meetings with family medicine trainees. This study wanted to explore the attitudes and opinions of regional coordinators and family medicine trainees concerning this new method.

**Methods:**

This was a qualitative study based on focus groups. The participants were regional coordinators and family medicine specialist trainees. The data were analysed based on the principles of thematic content analysis with inductive technique.

**Results:**

The study revealed five themes which were the same for the analysis of transcripts of both regional coordinators and family medicine trainees: 1) Meetings with trainees; 2) Coordination; 3) Characteristics of regional coordinators; 4) Position of regional coordinators, and 5) Evaluation of regional coordinators.

**Conclusion:**

Participants of the study have many expectations for this new programme. They expect progress in trainees’ clinical knowledge through experience-based group learning and with the help of the tutorship role of regional coordinators. The role of regional coordinators represents a new possibility for solving problems in the training programme in their coordinating role. In future, they have the potential to develop into an expert body that supervises the quality of training. A close follow-up is necessary to see if the position of regional coordinators is adequate and if they meet the expectations of the trainees as well as their own goals. Administrative and financial support for the programme is necessary. The project is important also in enabling the adaptation of the training programme’s needs and the regional characteristics of medical care.

## Background

In Slovenia, specialist training and the final specialist exam are compulsory for licensed independent work as family physicians with their own list of patients. Therefore, family medicine training takes place as a 4-year course and consists of two parts of rotations: in clinical specialties (mainly in hospitals) and in family medicine practices. During rotation in family medicine practices, trainees also have to attend compulsory theoretical modules on various clinical and organisational themes. Each family medicine specialist trainee is supervised by an advisor (head of training) during the whole training and by the immediate supervisor appointed for each rotation [1]. The training programme is led by the Medical Chamber of Slovenia, which appoints one national coordinator per specialisation. A national call for specialisation is published twice per year on the web site of the Medical Chamber and announced in daily newspapers. Most of the medical graduates start their chosen specialisation immediately after completing the internship, which lasts six months. The number of specialisation posts is limited according to the region and defined according to the agreement between the planned regional needs in health institutions and the Ministry of Health. Therefore, some candidates have to apply several times before they are accepted.

In the last year, a project has started that established a new role for several regional coordinators in the hierarchic level between the national coordinator and the advisors. To be recognised as a regional coordinator, the person has to be an experienced advisor and general practitioner, and must have teaching experience. The number of regional coordinators was determined by the number of trainees in the region. Ten to twelve trainees were allocated to each regional coordinator and six group meetings per year—starting in 2015—were planned. The purpose of these meetings is to help specialist trainees in their professional and personal development, but it is not specified in detail. The regional coordinator organises, coordinates and guides meetings while active work is expected to be done by the specialist trainees themselves. One of the trainees is in charge of the notes and minutes: attendance, topics discussed, findings, unresolved dilemmas and the agenda for the following meeting. Because the education process is performed in small groups and the topics of interest are student/trainee driven, the chosen prevailing teaching method was problem-based and case-based learning, both enabling active learning through the experience of problem solving, self-learning and collaboration between group members. Despite the lack of firm evidence for superiority in the learning outcome, this teaching method represents enjoyable and motivating approach to medical education [2]. Appropriate suggested topics were analysis of interesting or complex clinical cases, solving clinical vignettes, solving ethical dilemmas, analysis of medical errors, managing sick leave in practice and other.

In other countries, especially in the United Kingdom, similar teaching methods for family medicine trainees are already established. Coordinators are called training supervisors and they have a similar position as our regional coordinators in the specialisation process of family medicine [3]. Research shows that trainees accept this form of education very well [3]. The coordinators’ usefulness is seen especially in teaching structured case-management arising from practice, based on the European competencies of family medicine [4–6]. In the UK, this form of education is carried out by the individual teaching method (one-to-one).

In Slovenia, this is a new project, introduced only in family medicine training and not in other, clinical specialist training. To our knowledge, this is also a unique approach as it combines not only a teaching method but also additional tutoring for a small group of trainees in specialty training.

As such, the exact scope of this project is not well defined and expectations from both sides—the family medicine trainees and the regional coordinators—are not known. Therefore, we organised focus groups comprised of trainees and moderators and asked them similar questions to find out their expectations from the project of regional coordinators and the role of regional coordinators in the very early stage of the project where only limited experience has been obtained.

## Methods

A qualitative study with focus groups was designed to explore the opinion about the project idea, the opportunity of regional coordinators in improvement of the training programme according to the needs, the expected personal characteristics of the coordinators, the need for support in the project and possible evaluation methods of the project. Focus groups were organised with regional coordinators and with family medicine trainees separately.

### Participants

#### Regional coordinators

Out of ten regions with 31 regional coordinators, representatives of regional coordinators of various ages and both sexes from all Slovenian regions were invited, with at least one from a smaller region and up to three from the biggest regions. The sampling was purposive. Only two invited persons refused to participate due to unavailability to attend the meetings. Regional coordinators were invited both with and without academic backgrounds.

#### Family medicine trainees

Trainees in various levels of their specialisation were invited—beginners and those just before their specialist exam. Young specialists of up to 5 years after having completed the specialist exam were also invited. The participants were sampled from all Slovenian regions and worked in urban or rural practices.

Demographic characteristics of the participants are presented in Table 1.

**Table 1:** Demographic characteristics of the participants of the study

Focus groups	No. of focus groups	No. of participants	Age (mean range)	Gender (women)	Location of focus groups	Academic background	Location of the practice^a^ (urban)	Works in solo practice	Length of training^b^ (first half)
Regional coordinators	2	15	51.5 (42–63)	12	Strunjan Ljubljana	7	5	n/a	n/a
Specialist trainees	3	16	34.3 (27–34)	11	Maribor Velenje Kočevje	n/a	10	5	9

*n/a* not applicable

Legend:

^a^(urban > 30.000 inhabit.)

^b^number of trainees within the first half of training programme

Altogether, five focus groups were held at different locations throughout the country. We planned to organise three to five focus groups of trainees—the exact number was not fixed in advance, but depended on data saturation, which happens when additional data do not bring new information to the research question. This was reached with the fifth focus group.

### Progress of the study

After an initial explanation of the purpose of the study, the participants engaged in the discussion on open-ended questions for the semi-structured group interview. Basic demographic characteristics and years of experience were collected at the beginning (Table 1). Each group was moderated by an experienced person in qualitative research and observed by an observer. The interviews were audio-taped. The first evaluation between the moderator and the observer followed at the end of the first focus group, which led to small modifications in the set of questions for the following groups. The basic questions were the same for the regional coordinators and the trainees, and represented the framework within which the moderator could pose additional questions to explore the problem in more depth.

1. How did you accept the implementation of regional coordinators into specialist training?
2. Which weaknesses and needs in the specialist training programme can regional coordinators fulfil? Which competences could be improved by their implementation?
3. What are the essential positive characteristics of a regional coordinator (knowledge, skills, personal traits)? What traits would make a bad coordinator?
4. How could the project be evaluated?
5. A question posed only to regional coordinators was: what additional education, knowledge and skills would you need for this new role in the training programme?

### Analysis

All audiotapes were transcribed verbatim and checked for accuracy. The transcript was analysed using thematic analysis, which contains analysis of raw data by open coding, category formation and abstraction [7, 8] by the so-called inductive technique from text level.

The unit of analysis was defined, (a transcript of one focus group) and the unit of coding (raw data as blocks of quotations). Codes were assigned to the quotations. Three independent researchers coded the text (D.P., Z.K.K., B.P.) and in case of differences, discussed the analysis until an agreement was reached. In the second stage, duplicated codes and codes which were not relevant to the research question were reduced to a final list of codes. Consequently, the codes were grouped by their similarities into themes. Themes and codes of regional coordinators and specialist trainees were compared to each other. The last stage represented Interpretation of the analysis and was made by associating the text and the context of the codes and themes. Codes were assigned by appropriate quotations and interpreted in the context of the text.

## Results

The three focus groups of trainee analysis revealed 90 codes. Two groups of regional coordinators revealed 113 different codes altogether. The final result of the analysis revealed five themes, which were the same for both the analysis of transcripts of regional coordinators and family medicine trainees: 1. Meetings with trainees, 2. Coordination, 3. Characteristics of regional coordinators, 4. Position of regional coordinators and 5. Evaluation of regional coordinators (Table 2).

**Table 2:** Themes, categories and number of different codes (without repetition of codes) for family medicine trainees and regional coordinators

Themes	No of codes (trainees/RC)	Categories
Group work (dynamics?)	7/9	Meetings with specialist trainees
Topics of the meetings	10/7	
Organisational issues	7/3	
Connecting stakeholders	9/13	Work with coordination
Spec. training problem solving	10/7	
Personal characteristics	17/24	Characteristics of RC
Competence	12/13	
Personal gain for RC	0/6	
Problems of RC	8/17	Position of RC
Needs for development	3/8	
Evaluation of the project	7/7	Evaluation of project

### 1st category: meetings with family medicine trainees

1. Work in groups, group dynamics

Regional coordinators and family medicine trainees see many advantages of regular group meetings: possibilities of mutual help and communication, exchange of experience, group learning, comparison of solutions, group support and supervision. Regional coordinators also stated that the groups would help family medicine trainees to achieve a critical attitude towards themselves and help to get to know family medicine trainees within the region.

Citation1 *It could also be a support group, a method of supervision that we don’t have and we miss, because we all have problems, especially trainees.* (family medicine trainee, focus group 3).

Citation 2 *Especially so that we can talk about anything. And that it is confidential. We all have many mistakes and dilemmas, but it could all be easily solved. And maybe sometimes we do not dare ask during theoretical lectures, while here it is more intimate.* (family medicine trainee, focus group 2).

Citation 3 …*the opportunity for critical distance to their work, because some people in my group were quite self-centred and confident in their own right.* (regional coordinator, focus group 2).

Citation 4 …*the opportunity to see the different ways of working and that they also receive some incentive which they could do differently themselves.* (regional coordinator, focus group 1).

Citation 5 *Similar problems in the same environment.* (regional coordinator, focus group 1).

2. Content of the meetings

Family medicine trainees expect education and problem solving: how to manage the most common or/and difficult clinical problems, administrative rules, addressing special themes (such as burnout), ethical dilemmas, information about trainee payment and specialist training.

Regional coordinators were more general in describing the content of the meetings: adaptation to the needs of family medicine trainees, preparation for the specialist exam, themes for professional development, solving problems with mentors. They stated that the meetings should have formal but also informal content.

Citation 6 *So that each person would bring the cases from the practice we agree on, for example: administration today, professional themes the next time.* (family medicine trainee, focus group 1).

Citation 7 *At the beginning, we could initially focus on professional development that would be as individually adapted as possible and perhaps to the needs of the region.* (regional coordinator, focus group 1).

3. Organisational aspects of the meetings

Family medicine trainees and regional coordinators expect to receive the work plan and timetable of the meetings in advance. They expect the meetings to be a part of their regular work. Family medicine trainees expect reimbursement of travel expenses and refuse to have extra obligations or preparations for the meetings.

Citation 8 *I think it is right that the group meetings take place during our regular work time, I made a schedule of meetings for half a year or nine months in advance.* (regional coordinator, focus group 2).

### 2nd category: coordination

1. Connecting stakeholders

The regional coordinator is seen by family medicine trainees as a person who connects family medicine trainees, family medicine specialists, clinical specialists (mentors in clinical rotations). He represents the link to a national coordinator and should communicate with the main advisor. Regional coordinators should be connected amongst each other, embedded in the network and should together develop into an advisory body for training.

Regional coordinators also emphasised the connection with the institutions: Medical Chamber, departments of Family medicine, clinics involved in clinical rotations and being the intermediate person who transmits information from the family medicine trainees to other stakeholders.

Citation 9 *We could communicate with main supervisors and make proposals if the trainees pointed that out, and the same with immediate mentors in hospitals.* (regional coordinator, focus group 1).

2. Problem solving in specialist training

Family medicine trainees saw an opportunity in the regional coordinators’ knowledge of the region and the institutions for clinical rotations, knowing the programme of specialist training, and identifying the opportunities to improve it. They emphasised the problems in clinical rotations where regional coordinators could provide better quality by negotiating with the clinics. Help with an e-specialist training chart and exchanges abroad were also mentioned.

Furthermore, regional coordinators saw their task of problem solving in specialist training in general—especially in different regions, particularly in clinical rotations.

Citation 10 *He (regional coordinator) will provide a peaceful course of your specialisation, without exposing you, he will be a neutral person who will communicate with your direct supervisor or someone else at the clinical rotation.* (family medicine trainee, focus group 3).

Citation 11 *Maybe it’s really better that someone from the region, in a position of regional coordinator, tries to solve problems and, of course, if it is not possible he suggests further steps.* (regional coordinator, focus group 1).

### 3rd category: characteristics of RC

1. Regional coordinator as a person

Several personal characteristics were expected from regional coordinators: being supportive, having authority, a relaxed attitude to family medicine trainees, a fair relationship to family medicine trainees, non-confrontational, open-minded, not resentful. Family medicine trainees want regional coordinators to be continuously available for contacting, to be responsive, to give specific answers, to guide them but not to burden them or evaluate them.

Regional coordinators also stated that they should have a genuine interest in family medicine trainee and a friendly approach. They see themselves as a role model, a hard worker, demanding towards themselves, encouraging affiliation to family medicine, having a realistic opinion of family medicine trainees and with the ability to expose themselves for the family medicine trainees wellbeing.

Citation 12 *A person who is experienced, and is up to date, not that he works “a hundred years” and has no idea where the medicine went; that is open, communicative, has a desire to help, that he is a good organiser, able to quickly solve something, operational*. (family medicine trainee, focus group 3).

Citation 13 *I see myself as a tutor.* (regional coordinator, focus group 2).

Citation 14 *That is, in order to promote trainees to use their competences and not directly resolve their problems, but to empower them to know how to do it; to help them focus correctly and clarify what their opportunities and rights really are; that he wouldn’t interfere directly because it is not good.* (regional coordinator, focus group 1).

Citation 15 *That (being a regional coordinator) forces me to maintain “freshness” and personal professional development, and in fact, I do something for myself by giving forward to others.* (regional coordinator, focus group 2).

2. Competencies

Regional coordinators should have several competencies and skills: to be a good organiser, able to solve problems, have a good and updated clinical knowledge, be a good teacher and good in communication (adviser, listener, negotiator, and supportive).

Regional coordinators are looking for additional competencies for their role: skills to give/advice in psychological help for family medicine trainees, professional help, to know how to motivate and activate family medicine trainees, be a mediator, know the situation of the family medicine profession.

Regional coordinators also see the benefits for themselves: being a regional coordinator is an upgrade of being a mentor, it is an investment in themselves (their own competencies), and also a duty.

Citation 16 *You try to be as objective as possible, even when some problems arise, to know, I would say, the placement of our profession in the system and among other specialties. To know how to properly focus; of course regarding communication you should be partly a mediator; not only facilitator, but also the one who is able* – *if necessary* – *to talk to our colleagues, somewhat positively draw attention to some things; in short, additional determination, courage and, of course, additional skills in communicating with colleagues.* (regional coordinator, focus group 2).

Citation 17 *I think this is an upgrade of mentoring work* – *these regional coordinators.* (regional coordinator, focus group 2).

### 4th category: position/enforcement of regional coordinators

1. Problems of regional coordinators

Family medicine trainees saw problems in the unreliability of the project itself, unclear competencies of regional coordinators and doubt in the power of regional coordinators.

Regional coordinators also stated a negative attitude of family medicine trainees toward them/the project, low awareness and low responsiveness of family medicine trainees about the project, unclear expectations from them, and an undetermined financial reward for their work.

Citation 18 *I do not even know exactly what we should do* …(family medicine trainee, focus group 1).

Citation 19 *I was a little disappointed because I was receiving emails whether it is mandatory or if the Department (of family medicine) has a list (of attendees), or “will I be negatively considered, if I do not come, or will I get bonus points for the specialist examination if I attend*”? (regional coordinator, focus group 1).

Citation 20 *That communication and contact with supervisors (main advisors and advisors), highlighted by R. is my dilemma too, because in fact the competencies or responsibilities are not determined.* (regional coordinator, focus group 1).

2. Needs for the improvement of the project

Family medicine trainees emphasised the need for better information, integration, for a clear defining of the role of regional coordinators and for better organisational conditions (place for meetings).

Regional coordinators also stated the need for a better definition of their role and competencies, instruction for their work, better communicational skills, and connections with local health centres and their managers.

Citation 21 *All these regional coordinators should get together at the Medical Chamber and report what the problems are and what can be done and arrange these matters, which are difficult.* (family medicine trainee, focus group 3).

Citation 22 *In addition to communication, we certainly need more precise instructions on what to actually do.* (regional coordinator, focus group 1).

### 5th category: evaluation of regional coordinators and the project

In the answer on how to evaluate the project, family medicine trainees suggested that the activities and their own engagement, the number of solved problems and fulfilled expectations represent an informal evaluation. They suggested a questionnaire as a formal evaluation.

Regional coordinators stated that the participation of family medicine trainees at meetings, activities and notes from the meetings will show the successfulness of their work. They also suggested getting feedback from family medicine trainees in informal discussions and by formal, anonymous questionnaires.

Citation *23 I think that they (family medicine trainees) should tell themselves whether they received additional knowledge, skills, whether they were satisfied with such meetings.* (regional coordinator, focus group 2).

## Discussion

This study considered the new developed teaching method implemented in family medicine training in Slovenia and revealed two aspects of the new programme: regional coordinators as the new professional and personal leaders of trainees, and the regional aspect—addressing specifics and problems within the region where the trainees will work. Moreover, other opportunities for the regional coordinator role have been revealed, such as forming an expert consultation body for the development of Family medicine training.

The principle of regional coordinators working in specific regions results in the expectations to sensitively approach specific problems within the specific region and to improve regional problem solving. Their personal “role model” and coaching is coupled with their clinical and coordinative work with family medicine trainees. The results of the study show that there are some uncertainties about the role of regional coordinators, their competencies and the follow-up of the project, but nevertheless the expectations from the project and from regional coordinators are quite high.

The coordinators will work with groups of trainees. The expectations of regional coordinators and family medicine trainees are that this will be the opportunity to solve individual problems linked to specialist training, similar to other countries, where supervisors apply the one-on-one method. In addition, family medicine trainees will benefit from group dynamics, learn from others and receive peer feedback. The regional coordinator is also responsible for the personal development of family medicine trainees, and groups could be the setting where family medicine trainees develop a critical attitude towards themselves in a safe environment. They also emphasised the importance of the family medicine trainees of the same region to meet and create a social network, which is, according to some sources, a protective means to evade burnout [9]. To some extent, similar group work could be found in other training programmes. Rodnick reports on regional courses that are organised in the UK, led by regional trainers and the regional postgraduate office, aimed at in-depth learning of specific topics from an expert faculty teacher [10]. Cate reported about problem-based small group teaching in residency programmes in the Netherlands. He emphasised that this type of education should be performed from the student-centred perspective and that guiding students in the right direction should be more effective than transmitting knowledge—a similar opinion was stated by our regional coordinators [11].

Both groups (regional coordinators and family medicine trainees) stated similar expectations regarding the professional content of the meetings—discussing interesting clinical cases, ethical dilemmas and health insurance rules, but regional coordinators emphasised flexibility of themes according to family medicine trainees’ interests and suggestions, and they were less specific about the content. It seems that in this project, the personal link between the teacher, the trainees and the coordinating role is specific and unique. Nevertheless, family medicine trainees were strongly against taking on additional obligations in preparing for the meeting, possibly due to the voluntary nature of this activity, which is not a part of the official curriculum of specialist training. Both groups expect a high level of organisation of the meeting, which should take place during their regular work and should be planned well in advance. The advantage of a protected time off-site is also mentioned in Rodnick’s paper [10].

The second important opportunity of this project is seen in the coordinating role of the regional coordinators. The regional coordinator is perceived as the middle person between family medicine trainees and their advisor/immediate supervisors, between family medicine trainees and the national coordinator, between family medicine trainees and clinics on a secondary level where they rotate according to the training programme. The most accentuated was the need for coordination with clinics: during second level rotations, family medicine trainees attributed the majority of the problems to the inadequate implementation of their appointed training programme. The regional coordinators are expected to know specific regional problems better than the national coordinator and should be able to suggest solutions as well as assist in providing them. Despite these expectations, some of the family medicine trainees and regional coordinators were not confident that the regional coordinators possess the required competencies to solve such issues. Regional coordinators questioned the power and executive competencies of their coordinating function and stated that they would require better-defined positions in the training process.

Another suggestion was that regional coordinators should form a committee, which would be an advisory body in further development of the training and its quality. Similar advisory bodies for family medicine specialisation are already established in many other countries [11, 12].

There were many codes related to expected specific personality traits and various skills that regional coordinators should have. The expectations reflect the new demands for training of family physicians who need coaching, career advice, assistance and partnership in their development [13, 14]. Interestingly, this personal and individual approach is sought not only from their supervisors with whom they work one-to-one, but also from the regional coordinator. They should be supportive, open-minded and well intentioned, non-judgmental, fair and have a relaxed, non-resentful attitude towards family medicine trainees. Regional coordinators also stated the need for excellent personal traits—they should have high demands towards themselves, be hard-working and act as role models for family medicine trainees. As for competencies, besides having a good and updated clinical knowledge, pedagogical skills and organisational skills, they emphasised the importance of having good communication skills. This is important in the sense that they are expected to be good negotiators (in regards to clinics), mediators, capable of psychological support, advisors and motivators. Regional coordinators see their competencies in the light of their own professional experience, and in the understanding of the problems and the profession itself. The importance of good communication was stated also in other studies, including the use of modern technologies in communication and teaching skills [15, 16].

Some problems with the projects were mentioned by family medicine trainees and regional coordinators, including the poorly defined and incomplete role and competencies of the regional coordinator and the stability of the project. Some of the regional coordinators expressed doubt in the usefulness and the effect of their own work and the unknown expectations of family medicine trainees and others. They were afraid that there might be unrealistic expectations and a negative attitude of trainees in regards to their role and activities. They feel that family medicine trainees are resentful of group work and afraid of additional duties. An important fact is also that the financial reward for regional coordinator work is not defined. They also suggested that each regional coordinator should be connected to the regional health centre and that the centre manager should be aware of and supportive towards the project, which is again reflecting the regionalist aspect of the project.

The project should be formally evaluated by the questionnaires for family medicine trainees, but also by the activity of the work itself—the number of meetings, the number of solved problems, and the attendance of the family medicine trainees.

### Strengths and limitations of the study

This study managed to include regional coordinators and family medicine trainees with different backgrounds and settings. We believe that saturation was reached due to several aspects of the study and analysis. According to Morse, saturation is defined as “…building of rich data within the process of inquiry, by attending to scope and replication, hence, in turn, building the theoretical aspects of inquiry” [17, 18]. Firstly, the inclusion criteria provided the participation of both categories of participants—regional coordinators and trainees, all with characteristics that were considered important for the aim of the study (for example: length of training, different regions of the country, location of the practice etc.). The duration of the discussion in each focus group was more than an hour and well-focused, which is a good sign of richness of the data.

The attention to the scope of the research topic represents the depth of the topic: we believe that the obtained results were not only about the simple, basic expectation content of group meetings, but also that other phenomena were presented: the opportunity of this project and regional coordinators, and dangers for a poorer performance in the future. The obtained categories were evident from the first three analysed focus groups, whereas the variation within the categories was rich. This enables the phenomenon to become stronger, evident, consistent and “mature” [17].

The attention to replication was represented by the opinions of the participants which were replicated to some extent and consequently the codes began to replicate in the last analysed groups. At the end—the development of theoretical conceptualisation was a sign of richness of the data within the category.

#### The limitations of the study

The findings of this study can therefore be regarded as valid and reliable. Still—the limitation of this study might be the fact that only family medicine trainees motivated for this kind of teaching participated in the study. As a result, some themes might have been overlooked. The second issue is the early beginnings of the project and the limited experience of the trainees and regional coordinators. Therefore, the topic might be re-evaluated after the first year of its implementation. The study is the first part of a mixed methods research for the evaluation of the project. In the second part, we intend to design further strategies for the project evaluation. We had planned to develop a questionnaire, but it seems that another qualitative part would be necessary in evaluating the project after the first year of running.

## Conclusion

Family medicine is the first and, at the moment, the only specialty in Slovenia to implement several regional coordinators within each region in the training programme. It also has the largest number of specialist trainees. Their training programme is based mostly in the region where they are doing their specialisation. It was therefore assumed that the needs and problems differ to some extent according to the region where the trainees work. The results of our research confirmed this.

Expectations from the project that implements regional coordinators into the specialist training programme include several options of group learning, such as focussed curricular learning, problem-based learning, case studies and non-clinical issues for everyday practical work. Other important aspects are collegial support and the coordination role of regional coordinators (mostly) in problems of clinical rotations. Regional implementation of the training programme generates specific problems, which are expected to be addressed by regional coordinators. To be successful, the project needs a more specific purpose and goals, such as competencies in the coordination role of regional coordinators, its evaluation and also support via financial and administrative means. It is hopeful that this research will prove to the Medical Chamber that the project is an important means for quality improvement of the training programme and that they will consider providing financial support. The unexpected opportunity of regional coordinators developing into a quality expert group in specialist training has been suggested as a result of this analysis. It is not known if other specialisations in the country will follow our lead, as this depends on the financial resources as well. Nevertheless, we give an example where the regional, pedagogical and coordinative structure is welcomed and needed by the trainees.

## Ethics approval and consent to participate

Ethical approval for the study was granted by the Republic of Slovenia National Medical Ethics Committee under no. 66/02/15. All participants gave informed consent to their participation in the study.

## Consent for publication

No potentially identifying details are included in the text. Consent to publish is not applicable for this manuscript.

## Availability of data and materials

The dataset supporting the conclusions of this article is included within the article. The full data are available in audio format and as transcripts of all focus groups in Slovenian language. Under request they are available by the corresponding author.
